# Supplementary material for: Organ-Specific and Mixed Responses to Pembrolizumab in Patients with Unresectable or Metastatic Urothelial Carcinoma: A Multicenter Retrospective Study
Source: Cancers (Basel). 2022 Mar 29;14(7):1735. doi: 10.3390/cancers14071735 (PMC8997142; doi:10.3390/cancers14071735)
Supplement: Supplementary file 1 [file cancers-14-01735-s001.zip › cancers-1661821-supplementary.pdf]

Table S1. Univariate and multivariate cox regression analyses of clinicopathological factors for overall survival.

Variables		Overall survival					
		Univariate analysis			Multivariate analysis		
		**HR**	**95% CI**	***P* value**	**HR**	**95% CI**	***P* value**
Age	< 75	1		*0.62*			
	≥ 75	1.13	0.70-1.82				
Sex	Male	1		*0.87*			
	Female	0.95	0.55-1.66				
ECOG-PS	0,1	1		0.0003*	1		0.012*
	≥ 2	5.53	2.20-13.9		2.29	1.20-4.36	
Hemoglobin (g/dL)	≥ 10	1		*0.29*			
	< 10	1.33	0.79-2.25				
eGFR (mL/min/1.73m2)	≥ 45	1		*0.50*			
	< 45	1.18	0.73-1.93				
Primary Site	BC	1		*0.52*			
	UTUC	0.86	0.54-1.37				
Variant historogy	No	1		*0.62*			
	Yes	0.62	0.34-1.90				
Number of prior chemotherapy	1	1		*0.92*			
	≥ 2	1.03	0.58-1.83				
Interval since last chemotherapy	< 3	1		*0.28*			
(month)	≥ 3	0.76	0.47-1.24				
Local recurrence	No	1		*0.67*			
	Yes	0.87	0.45-1.67				
Local advance	-	1		*0.08*			
	+	1.63	0.94-2.83				
Lymph node metastasis	No	1		*0.50*			
	Yes	1.18	0.73-1.90				
Lung metastasis	-	1		*0.06*			
	+	1.6	0.99-2.60				
Liver metastasis	No	1		0.030*	1		0.21
	Yes	2.16	1.08-4.31		1.45	0.82-2.58	
Bone metastasis	No	1		0.010*	1		0.18
	Yes	2.36	1.23-4.55		1.47	0.84-2.57	

HR=hazard ratio; CI=confidence interval; ECOG-PS = eastern cooperative oncology group-performance status; eGFR = estimated glomerular filtration rate; BC = bladder carcinoma; UTUC = upper tract urotherial carcinoma;
* = statisticallysignificant

MR	Local recurrence	Local advance	Lymph node	Lung	Liver	Bone	Brain	Pleura	Peritoneal dissemination	Others
1	-	-	-	-	-	-	PR	-	-	PD
2	-	-	PD	CR	-	-	-	-	-	PR
3	-	-	SD	PD	PR	-	-	-	-	-
4	-	PD	-	CR	-	-	-	-	-	-
5	-	-	PR	PD	-	-	-	-	-	-
6	-	-	PD	CR	-	-	-	-	-	-
7	-	-	PR	-	-	PD	-	-	-	-
8	-	PD	PR	PR	-	-	-	-	-	-
9	-	-	-	-	PR	SD	-	PD	PD	-
10	PR	-	PR	CR	PR	-	-	CR	CR	PD
11	-	PR	PD	PD	-	-	PD	-	-	-
12	-	-	-	PR	-	PD	-	-	-	-

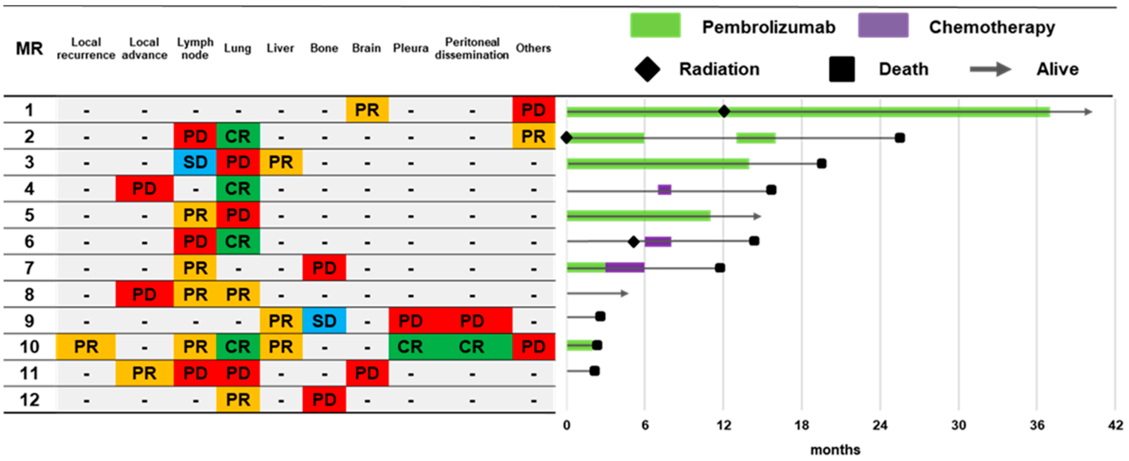

Figure S1. Organ-specific response of patients with MR and swimmer plot describing the clinical course after MR confirmation The table shows the organ-specific response of the 12 patients with MR. The swimmer plot shows the following: pembrolizumab (green bar), chemotherapy (purple bar), radiation therapy (black diamond), and death (black square). Arrows indicate that the patient is alive. MR = mixed response; CR = complete response; PR = partial response; SD = stable disease; PD = progression disease
